# F223. COMPARATIVE STUDY OF HEART RATE VARIABILITY AND EMOTIONAL RESPONSE TO POSITIVE AND NEGATIVE AUDIOVISUAL STIMULATION IN PATIENTS WITH CHRONIC SCHIZOPHRENIA AND HEALTHY CONTROL

**DOI:** 10.1093/schbul/sby017.754

**Published:** 2018-04-01

**Authors:** Sang-Yeol Lee, Won-Myong Bahk, Young-Joon Kwon, Duk-In Jon, Moon Doo Kim, Beomboo Nam, Sung-Yong Park, Min-Kyu Song

**Affiliations:** 1 Wonkwnag University School of Medicine and Hospital; 2 Yeouido St. Mary’s Hospital, The Catholic University of Korea; 3 Soonchunhyang University Chunan Hospital; 4 College of Medicine, Hallym University;; 5 School of Medicine, Jeju National University; 6 School of Medicine, Konbuk University; 7 Keyo Hospital; 8 Yesan Mental Health Clinic

## Abstract

**Background:**

This study was to investigate the Heart Rate Variability(HRV) and emotional response to positive and negative audiovisual stimulation in patients with chronic schizophrenia and healthy control group

**Methods:**

Among 253 chronic schizophrenic patients admitted in 00 Hospital in 00 city by psychiatrist, 104 patients were informed about this research and consented. Those who met this study criteria were randomly selected. 35 healthy control consisted of peoples that did not have past and present history of mental and physical illness. Positive and negative affect and HRV were compared between chronic schizophrenia and healthy control groups, and positive and negative affect and HRV to positive and negative audiovisual stimulation were measured according to planed research process. Positive and negative audiovisual stimulation was defined by an art therapy professionalist and a psychiatrist as 10 positive and negative pictures. 3 positive and negative musics were shown to two groups for 4 minutes simultaneously. Positive and negative audiovisual stimulation were shown to two groups during 1-week intermission. HRV was measured with Ubpulse H3, an equipment for autonomic nervous system test made by Laxtha company and also analyzed by frequency domain analysis. Emotional Empathy Scale(EES) and Positive Affect and Negative Affect Schedule (PANAS) of two groups were measured at baseline and after positive and negative audiovisual stimulation. Global Assessment of Functioning Scale(GAF) and Positive and Negative Syndrome Scale(PANSS) of chronic schizophrenia group were measured by a psychiatrist.

**Results:**

1) Positive affect of patients group were significantly lower than control group, negative affect of patients group were significantly higher than control group. Low Frequency(LF), High Frequency(HF), and Total Power(TP) of HRV in patients group were significantly lowered than control group at baseline.

2) 7 subscales of emotional empathy scale were lowered in patients group compared to control group.

3) Positive affect of patients group was significantly less increased compared to the control group after positive audiovisual stimulation, negative affect of patients group was significantly less decreased to the control group after positive audiovisual stimulation.

4) Positive affect of patients group was increased after negative audiovisual stimulation, but positive affect of control group was significantly decreased compared to the patients group after negative audiovisual stimulation. There was no significant difference in negative affect between two groups after audiovisual stimulation.

5) LF of patients group was significantly higher than control group after positive audiovisual stimulation, HF and TP of patients group were significantly lowered than control group after positive audiovisual stimulation.

6) LF of patients group was significantly higher than control group after negative audiovisual stimulation, HF and PT of patients were significantly lowered than control group after negative audiovisaul stimulation.

**Discussion:**

Patients with schizophrenia showed lower positive affect, higher negative affect, and lowered HRV parameters compared to the control group. They also showed lower empathy ability and inappropriate and non-contexual response. Schizophrenic patients represented hypersensitive sympathetic nervous system activity and lowered parasympathetic nervous system activity to the audiovisual stimulation. These results suggested that schizophrenic patients would show higher negative affect, less adaptive autonomic nervous system and hypersensitive or sharp to audiovisual stimulation, and decreased relaxation ability after stimulation. Audiovisual stimulation in integrative arts therapy program for schizophrenia might have avoid overactive sympathetic stimulation and recommend activate parasympathetic stimulation. Integrative art therapy for schizophrenia must be sufficiently relaxed, empathetic, and promote positive affect during therapeutic process.

